# Information Measures for Generalized Order Statistics and Their Concomitants under General Framework from Huang-Kotz FGM Bivariate Distribution

**DOI:** 10.3390/e23030335

**Published:** 2021-03-12

**Authors:** Mohamed A. Abd Elgawad, Haroon M. Barakat, Shengwu Xiong, Salem A. Alyami

**Affiliations:** 1 School of Computer Science and Technology, Wuhan University of Technology, Wuhan 430070, China; xiongsw@whut.edu.cn; 2 Department of Mathematics, Faculty of Science, Benha University, Benha 13518, Egypt; 3 Department of Mathematics, Faculty of Science, Zagazig University, Zagazig 44519, Egypt; hbarakat@zu.edu.eg; 4 Department of Mathematics and Statistics, Faculty of Science, Imam Mohammad Ibn Saud Islamic University (IMSIU), Riyadh 13318, Saudi Arabia; saalyami@imamu.edu.sa

**Keywords:** concomitants, dual generalized order statistics, Huang–Kotz FGM family, Shannon’s entropy, Fisher information number

## Abstract

In this paper, we study the concomitants of dual generalized order statistics (and consequently generalized order statistics) when the parameters $\gamma_{1},\ldots ,\gamma_{n}$ are assumed to be pairwise different from Huang–Kotz Farlie–Gumble–Morgenstern bivariate distribution. Some useful recurrence relations between single and product moments of concomitants are obtained. Moreover, Shannon’s entropy and the Fisher information number measures are derived. Finally, these measures are extensively studied for some well-known distributions such as exponential, Pareto and power distributions. The main motivation of the study of the concomitants of generalized order statistics (as an important practical kind to order the bivariate data) under this general framework is to enable researchers in different fields of statistics to use some of the important models contained in these generalized order statistics only under this general framework. These extended models are frequently used in the reliability theory, such as the progressive type-II censored order statistics.

## 1. Introduction

In testing the strength of materials, reliability analysis, lifetime studies, etc. the realizations of experiments arise in nondecreasing order and, therefore, we need to consider several models of ascendingly ordered random variables (RVs). Theoretically, many of such models, such as ordinary order statistics, order statistics with non-integral sample size, sequential order statistics, record values, Pfeifer’s record model and progressive type-II censored order statistics (POSs), are contained in what is known as the generalized order statistics (GOSs). The concept of the GOSs was introduced by [1] as a unified approach to these aforesaid models. The concept of GOSs enables a common approach to structural similarities and analogies between several models of ascendingly ordered RVs. Well-known results can be subsumed, generalized, and integrated within a general framework. Therefore, the concept of GOSs provides a large class of models with many interesting, important and useful properties for both the description and the analysis of practical problems.

The dual GOSs (DGOSs) was introduced by [2] as a parallel concept of GOSs to enable a common approach to descendingly ordered RVs. The DGOSs contain many important models of ordered RVs such as reversed order statistics, lower *k*-records and lower Pfeifer’s records. Burkschat et al. [2] has shown that (cf. Theorem 3.3) there is a direct link between DGOSs and GOSs. Therefore, for any result in the model of DGOSs, there exists a corresponding one in the GOSs model. Consequently, all the results of this paper can be easily transformed into the GOSs model. Let F(.) be an arbitrary continuous distribution function (DF) with probability density function (PDF) f(.). Then, the RVs $X_{d}\left(1,n,\tilde{m},k\right)\geq X_{d}\left(2,n,\tilde{m},k\right)\geq \ldots \geq X_{d}\left(n,n,\tilde{m},k\right)$ are said to be DGOSs if their joint probability density function (JPDF) is given by (cf. [2])

$$f_{1,\ldots ,n:n}^{d(\tilde{m},k)}\left(x_{1},\ldots ,x_{n}\right)=\left(\prod_{j=1}^{n}\gamma_{j}\right)\left(\prod_{j=1}^{n-1}F^{\gamma_{j}-\gamma_{j+1}-1}\left(x_{j}\right)f\left(x_{j}\right)\right)F^{k-1}\left(x_{n}\right)f\left(x_{n}\right),$$

where $F_{}^{-1}\left(1\right)\geq x_{1}\geq \ldots \geq x_{n}\geq F_{}^{-1}\left(0\right).$ The parameters $\gamma_{1},\ldots ,\gamma_{n}$ are defined by $\gamma_{n}=k>0,\gamma_{r}=k+n-r+\sum_{j=r}^{n-1}m_{j},r=1,\ldots ,n-1,\;\mathrm{and}\;\tilde{m}=\left(m_{1},m_{2},\ldots ,m_{n-1}\right)\in R.$

In this paper, we assume that the parameters $\gamma_{1},\ldots ,\gamma_{n}$ are pairwise different, i.e., $\gamma_{i}\neq \gamma_{j},i\neq j,\;i,j=1,2,\ldots ,n-1.$ With this assumption, we get a very wide subclass of DGOSs, which contains the reversed *m*-DGOSs (*m*-DGOSs model is an important subclass of DGOSs, for which $m_{1}=m_{2}=\ldots m_{n-1}=m$), the reversed order statistics and the lower Pfeifer’s records. For this general subclass of DGOSs, the PDF of *r*th DGOS, 1≤r≤n, and the JPDF of *r*th and *s*th DGOSs, 1≤r<s≤n, respectively, are given by

(1) $$f^{X_{d(r,n,\tilde{m},k)}}\left(x\right)=C_{r-1}\sum_{i=1}^{r}a_{i}\left(r\right)F^{\gamma_{i}-1}\left(x\right)f\left(x\right),\;\;x\in R,$$

and

(2) $$f^{X_{d(r,n,\tilde{m},k)},X_{d(s,n,\tilde{m},k)}}\left(x,y\right)=C_{s-1}\left[\sum_{i=r+1}^{s}a_{i}^{\left(r\right)}\left(s\right){\left(\frac{F(y)}{F(x)}\right)}^{\gamma_{i}}\right]\left[\sum_{i=1}^{r}a_{i}^{}\left(r\right)F^{\gamma_{i}}\left(x\right)\right]\frac{f(x)}{F(x)}\frac{f(y)}{F(y)},x>y,$$

where Cr−1=∏i=1rγi,ai(r)=∏j=1j≠ir1γj−γi,1≤i≤r≤n, and ai(r)(s)=∏j=r+1j≠is1γj−γi,r+1≤i≤s≤n (cf. [2,3]), respectively. For more detail about this general subclass, see [4,5,6].

Morgenstern [7] introduced Farlie–Gumble–Morgenstern (FGM) bivariate distribution for the Cauchy marginals. Later, [8] studied FGM for exponential distribution. Farlie [9] considered this family in the general form $F_{X,Y}\left(x,y\right)=F_{X}\left(x\right)F_{Y}\left(y\right)\left(1+\alpha \left(1-F_{X}\left(x\right)\right)\left(1-F_{Y}\left(y\right)\right)\right)$, −1≤α≤+1, where $F_{X}\left(x\right)=P\left(X\leq x\right)$ and $F_{Y}\left(y\right)=P\left(Y\leq y\right)$ are its arbitrary marginal DFs. The FGM family with general marginal DFs is a flexible family of bivariate DFs and valuable in many applications that are connected to data that exhibits low correlation.

In the last two decades, many authors have dealt with modifications of the FGM family allowing high correlation between its components. For example, see [10,11,12,13,14,15,16,17,18,19,20,21,22,23,24,25]. One of the most important and flexible generalizations of the classical FGM family was introduced by [26] by adding an additional shape parameter. The DF and PDF of the Huang and Kotz’s generalization (denoted by HK–FGM) are given by

(3) $$F_{X,Y}\left(x,y\right)=F_{X}\left(x\right)F_{Y}\left(y\right)\left[1+\alpha \left(1-F_{X}^{p}\left(x\right)\right)\left(1-F_{Y}^{p}\left(y\right)\right)\right],\;p>0,$$

and

$$f_{X,Y}\left(x,y\right)=f_{X}\left(x\right)f_{Y}\left(y\right)\left[1+\alpha \left(1-\left(1+p\right)F_{X}^{p}\left(x\right)\right)\left(1-\left(1+p\right)F_{Y}^{p}\left(y\right)\right)\right],\;p>0,$$

where $f_{X}\left(x\right)$ and $f_{Y}\left(y\right)$ are the PDFs of the RVs *X* and Y, respectively. The HK–FGM family (3) allows correlation higher than the FGM family (i.e., for p=1). The admissible range of values for the association parameter α in the family (3) is $-p^{-2}\leq \alpha \leq p^{-1}$ and the range between the maximal and minimal values of the correlation coefficient ρ is given by $-{\left(p+2\right)}^{-2}\min\left(1,p^{2}\right)\leq \rho \leq 3p{\left(p+2\right)}^{-2}.$

The concept of concomitants, which are also called the induced order statistics, arises when one sorts the members of a random sample according to corresponding values of another random sample. The general theory of concomitants of order statistics was originally studied by [27]. For a comprehensive review of some applications of the concept of concomitants of order statistics, see [28]. Let $(X_{i},Y_{i}),i=1,2,\ldots ,n,$ be a random sample from a bivariate DF $F_{X,Y}\left(x,y\right).$ If $X_{d}\left(r,n,\tilde{m},k\right)$ be the *r*th DGOS, then the *Y* value associated with $X_{d}\left(r,n,\tilde{m},k\right)$ is called the concomitant of the *r*th DGOS and denoted by $Y_{\left[r,n,\tilde{m},k\right]},r=1,2,\ldots ,n.$ The PDF of the concomitants of *r*th DGOS is given by (cf. [29,30])

(4) $$f_{\left[r,n,\tilde{m},k:p\right]}\left(y\right)=\int_{-\infty }^{\infty }f_{Y|X}\left(y|x\right)f^{X_{d}\left(r,n,\tilde{m},k\right)}\left(x\right)dx,$$

where $f^{X_{d}\left(r,n,\tilde{m},k\right)}\left(x\right)$ is the PDF of $X_{d}\left(r,n,\tilde{m},k\right)$ (defined by (1)) and $f_{Y|X}\left(y|x\right)$ is the conditional PDF of *Y* given X. More generally, for 1≤r<s≤n, the JPDF of the concomitants of *r*th and *s*th DGOSs is given by

(5) $$\begin{array}{c} f_{\left[r,s,n,\tilde{m},k:p\right]}\left(y_{1},y_{2}\right)=\int_{-\infty }^{\infty }\int_{-\infty }^{x_{1}}f_{Y|X}\left(y_{1}|x_{1}\right)f_{Y|X}\left(y_{2}|x_{2}\right)f^{X_{d}\left(r,n,\tilde{m},k\right),X_{d}\left(s,n,\tilde{m},k\right)}\left(x_{1},x_{2}\right)dx_{2}dx_{1}, \end{array}$$

where $f^{X_{d}\left(r,n,\tilde{m},k\right),X_{d}\left(s,n,\tilde{m},k\right)}\left(x_{1},x_{2}\right)$ is the JPDF of $X_{d}\left(r,n,\tilde{m},k\right)$ and $X_{d}\left(s,n,\tilde{m},k\right)$ (defined by (2)).

Shannon [31] proposed a measure of uncertainty or variability associated with a given RV, as a generalization of Boltzman–Gibbs entropy of classical statistical mechanics. Later, this measure was popularized in the name Shannon entropy. The Shannon entropy measures the quantity of knowledge gained, or removed uncertainty, by disclosing the value of a RV. In the literature, many studies about this measure can be found, e.g., [32,33,34].

The Fisher information (FI) number is the second moment of the “score function”, and it is an FI for a location parameter (cf. [35]). In recent years, the FI number has been frequently used in different aspects of science. For example, the FI number has been used to develop a unifying theory physical law called the principle of “extreme physical information”(cf. [36]).

In the literature, there are many authors that investigate the concomitants of GOSs whilst considering some information measures. Tahmasebi and Jafari [37] studied the FI number for concomitants of *m*-GOSs in the FGM family. Mohie El-Din et al. [30] studied the concomitants of GOSs from the FGM family, when $\gamma_{i}\neq \gamma_{j},i\neq j,\;i,j=1,2,\ldots ,n-1.$ Moreover, Mohie El-Din et al. [38] studied the Shannon entropy and the FI number for the concomitants of *m*-GOSs from FGM family for some special known marginals. Recently, Abd Elgawad et al. [10] studied the FI number for concomitants of *m*-DGOSs in the HK–FGM family. As a natural extension of the results obtained by [30,38], we study the concomitants of DGOSs (in the case when $\gamma_{i}\neq \gamma_{j},i\neq j,\;i,j=1,2,\ldots ,n-1$) from the HK–FGM model. Furthermore, the Shannon entropy and FI number of DGOSs from the HK–FGM model are investigated for some marginals, such as exponential, Pareto and power function distributions.

The distributional properties and some important information measures such as FI number and Shannon’s entropy of the concomitants of GOSs and DGOSs from some important extensions of the FGM model, such as the HK–FGM model, were recently studied in [10,11,12,13,14,15]. All of these studies were carried out for the submodels m-GOSs and m-DGOSs, which include many interesting models such as ordinary order statistics and sequential order statistics. However, a lot of practical important models contained in the families of GOSs and DGOSs are excluded from these submodels, e.g., the POSs. The POS model is an important method for obtaining data in lifetime tests, where live units removed early on can be readily used in other tests, thereby saving cost to the experimenter, and a comprise can be achieved between time consumption and observation of some extreme values. In this paper, we study the concomitants of GOSs and DGOSs from the HK–FGM model under a general set up including general important censoring models such as POSs.

The remainder of this paper is organized as follows. In Section 2, we study some important distributional characteristics, such as the marginal DF and some useful recurrence relations between moments for the single and product of the concomitants of the *r*th DGOS for HK–FGM model for any arbitrary marginal. Moreover, the concomitants of the POSs are studied for this model. Section 3 is devoted to studying the Shannon entropy for concomitants of DGOSs from the HK–FGM family. This measure is evaluated and studied for some well-known distributions such as the exponential, Pareto and power distributions. The study of the FI number is tackled in Section 4, where it is computed and studied for the exponential, Pareto and power distributions.

## 2. Concomitants of DGOs Based on HK–FGM

In this section, the marginal DF, moment generating function (MGF), moments and some recurrence relations between MGF and moments for single and product of concomitants of DGOSs for the HK–FGM model are obtained for any arbitrary marginals. Moreover, the concomitants of POSs are studied for the HK–FGM model.

### 2.1. Marginal Distribution of Concomitants

Let $X∼F_{X}$ and $Y∼F_{Y}.$ Then, the PDF given in (4) can be written in the form

(6) $$\begin{array}{cc} & f_{\left[r,n,\tilde{m},k:p\right]}\left(y\right)=\int_{-\infty }^{\infty }f_{Y|X}\left(y|x\right)f^{X_{d}\left(r,n,\tilde{m},k\right)}\left(x\right)dx\\ =\; & \;\;\;\;\int_{-\infty }^{\infty }f_{Y}\left(y\right)\left[1\;+\;\alpha \left(1-\left(1+p\right)F_{X}^{p}\left(x\right)\right)\left(1-\left(1+p\right)F_{Y}^{p}\left(y\right)\right)\right]\;\;\left[C_{r-1}\sum_{i=1}^{r}a_{i}\left(r\right)F_{X}^{\gamma_{i}-1}\left(x\right)f_{X}\left(x\right)\right]dx\\ =\; & f_{Y}\left(y\right)\left\{1+\left(1-\left(1+p\right)F_{Y}^{p}\left(y\right)\right)\Omega_{r,n,\tilde{m},k:p}\right\}=f_{Y}\left(y\right)+\Omega_{r,n,\tilde{m},k:p}\left[f_{Y}\left(y\right)-f_{V}\left(y\right)\right]\\ =\; & \left(1+\Omega_{r,n,\tilde{m},k:p}\right)f_{Y}\left(y\right)-\Omega_{r,n,\tilde{m},k:p}f_{V}\left(y\right), \end{array}$$

where $f_{V}\left(y\right)$ is the PDF of the RV $V∼F_{Y}^{p+1}$ and $\Omega_{r,n,\tilde{m},k:p}=\alpha \left[1-\left(1+p\right)C_{r-1}\sum_{i=1}^{r}\frac{a_{i}\left(r\right)}{\gamma_{i}+p}\right].$

**Remark** **1.** *By considering the well-known relation between the DGOSs and GOSs (cf. Theorem 3.3 in [2]) and by putting p=1 in (6), the marginal PDF of concomitants of GOS for FGM can be easily deduced, which was obtained by [30].*

By using (6), the MGF of $Y_{\left[r,n,\tilde{m},k\right]}$ is given by

(7) $$M_{\left[r,n,\tilde{m},k:p\right]}\left(t\right)=E\left[\exp\left(tY_{\left[r,n,\tilde{m},k\right]}\right)\right]=\left(1+\Omega_{r,n,\tilde{m},k:p}\right)\;M_{Y}\left(t\right)-\Omega_{r,n,\tilde{m},k:p}\;M_{V}\left(t\right),$$

where $M_{Y}\left(t\right)$ and $M_{V}\left(t\right)$ are the MGFs of RVs *Y* and V, respectively. Thus, by using (6) (or by using (7)), the *ℓ*th moment of $Y_{\left[r,n,\tilde{m},k\right]}$ is given by

(8) $$\begin{array}{c} \mu_{\left[r,n,\tilde{m},k:p\right]}^{\left(\ell \right)}=E\left[Y_{\left[r,n,\tilde{m},k\right]}^{\ell }\right]=\left(1+\Omega_{r,n,\tilde{m},k:p}\right)\;\mu_{Y}^{\left(\ell \right)}-\Omega_{r,n,\tilde{m},k:p}\;\mu_{V}^{\left(\ell \right)}, \end{array}$$

where $\mu_{Y}^{\left(\ell \right)}=E\left[Y^{\ell }\right]$ and $\mu_{V}^{\left(\ell \right)}=E\left[V^{\ell }\right].$ In general, if h(y) is a measurable function of y, then

$$E\left[h\left(Y_{\left[r,n,\tilde{m},k\right]}\right)\right]=\left(1+\Omega_{r,n,\tilde{m},k:p}\right)\;E\left[h\left(Y\right)\right]-\Omega_{r,n,\tilde{m},k:p}\;E\left[h\left(V\right)\right],$$

provided the expectations exist. The following theorem gives a useful general recurrence relation for $E[h\left(Y_{\left[r,n,\tilde{m},k\right]}\right)].$

**Theorem** **1.** *For any 2≤r≤n, we get*

$$E\left[h\left(Y_{\left[r,n,\tilde{m},k\right]}\right)\right]-E\left[h\left(Y_{\left[r-1,n,\tilde{m},k\right]}\right)\right]=\alpha p\left(1+p\right)C_{r-2}\sum_{i=1}^{r}\frac{a_{i}\left(r\right)}{\gamma_{i}+p}\left(E[h(Y)]-E[h(V)]\right).$$

**Proof.**  By using the easy-to-prove relations $a_{i}\left(r-1\right)=\left(\gamma_{r}-\gamma_{i}\right)a_{i}\left(r\right),\;C_{r-2}=\frac{C_{r-1}}{\gamma_{r}}$ and $\sum_{i=1}^{r}a_{i}\left(r\right)=0,$ we can write

$$\begin{array}{ccc} E[h\left(Y_{\left[r,n,\tilde{m},k\right]}\right)] & - & E\left[h\left(Y_{\left[r-1,n,\tilde{m},k\right]}\right)\right]=\left(\Omega_{r,n,\tilde{m},k:p}-\Omega_{r-1,n,\tilde{m},k:p}\right)\left(E[h(Y)]-E[h(V)]\right)\\ & = & \alpha \left(1+p\right)\left(C_{r-2}\sum_{i=1}^{r-1}\frac{a_{i}\left(r-1\right)}{\gamma_{i}+p}-C_{r-1}\sum_{i=1}^{r}\frac{a_{i}\left(r\right)}{\gamma_{i}+p}\right)\left(E[h(Y)]-E[h(V)]\right)\\ & = & -\alpha \left(1+p\right)C_{r-2}\sum_{i=1}^{r}\frac{a_{i}\left(r\right)\gamma_{i}}{\gamma_{i}+p}\left(E[h(Y)]-E[h(V)]\right)\\ & = & \alpha p\left(1+p\right)C_{r-2}\sum_{i=1}^{r}\frac{a_{i}\left(r\right)}{\gamma_{i}+p}\left(E[h(Y)]-E[h(V)]\right). \end{array}$$

 □

**Example** **1.** *Usually, in lifetime experiments, due to the restrictions of limited time and cost, accurate product lifetime data cannot be observed so we have censored data. One of most important censoring schemes is progressive type-II censoring. In this example, we consider the POSs with general censoring scheme $(R_{1},\ldots ,R_{n}).$ Let $F_{X,Y}$ be a continuous bivariate DF. Furthermore, let $X_{1:n}^{\tilde{R}},X_{2:n}^{\tilde{R}},\ldots ,X_{n:n}^{\tilde{R}}$ be POSs with general scheme $\tilde{R}=\left(R_{1},\ldots ,R_{n}\right),$ where $N=n+\sum_{i=1}^{n}R_{i}$ identical units are placed on a lifetime test. Bairamov and Eryilmaz [39] derived the PDF of the concomitant $Y_{\left[r:n\right]}^{\tilde{R}}$ of the POS $X_{r:n}^{\tilde{R}}$ by*

$$\begin{array}{c} f_{Y_{\left[r:n\right]}^{\tilde{R}}}\left(y\right)=C_{r-1}\sum_{i=1}^{r}\frac{a_{i}\left(r\right)}{\gamma_{i}}f_{Y_{\left[1:\gamma_{i}\right]}}\left(y\right), \end{array}$$

*where Cr−1=∏j=1rγj,ai(r)=∏j=1j≠ir1γj−γi,1≤i≤r≤n, the empty product $\prod_{\phi }$ is defined to be 1, $\gamma_{j}=N-\sum_{i=1}^{j-1}R_{i}-j+1,1<j\leq n,\gamma_{1}=N,$$f_{1:\gamma_{i}}\left(x\right)=\gamma_{i}{\left(1-F\left(x\right)\right)}^{\gamma_{i}-1}f\left(x\right)$ and $f_{Y_{\left[1:\gamma_{i}\right]}}\left(y\right)=\int_{-\infty }^{\infty }f_{Y|X}\left(y|x\right)f_{1:\gamma_{i}}\left(x\right)dx$ is the PDF of the concomitant of the minimum order statistics from a sample of size $\gamma_{i}.$ Now, let $F_{X,Y}$ be HK–FGM with uniform marginals. Then, $F_{X,Y}\left(x,y\right)=xy\left[1+\alpha \left(1-x^{p}\right)\left(1-y^{p}\right)\right],\;0\leq x,y\leq 1,$ and $f_{X,Y}\left(x,y\right)=1+\alpha \left(1-\left(1+p\right)x^{p}\right)$$\left(1-\left(1+p\right)y^{p}\right).$ Thus,*

$$f_{Y_{\left[1:\gamma_{i}\right]}}\left(y\right)=1+\alpha \left(1-\left(1+p\right)y^{p}\right)-\gamma_{i}\alpha \left(1+p\right)\left(1-\left(1+p\right)y^{p}\right)\beta \left(\gamma_{i},p+1\right).$$

*Therefore, the PDF of $Y_{\left[r:n\right]}^{\tilde{R}}$ is given by*

$$f_{Y_{\left[r:n\right]}^{\tilde{R}}}\left(y\right)=C_{r-1}\sum_{i=1}^{r}\frac{a_{i}\left(r\right)}{\gamma_{i}}\left\{1+\alpha \left(1-\left(1+p\right)y^{p}\right)-\gamma_{i}\alpha \left(1+p\right)\left(1-\left(1+p\right)y^{p}\right)\beta \left(\gamma_{i},p+1\right)\right\}.$$

### 2.2. Joint Distribution of Concomitants of DGOSs in HK–FGM Model

The following theorem gives the JPDF $f_{\left[r,s,n,\tilde{m},k:p\right]}\left(y_{1},y_{2}\right)$ (defined by (5)) of the concomitants $Y_{\left[r,n,\tilde{m},k\right]}$ and $Y_{\left[s,n,\tilde{m},k\right]},r<s,$ in the HK–FGM model for arbitrary marginals.

**Theorem** **2.** *For any 1≤r<s≤n, we have*

(9) $$\begin{array}{cc} f_{\left[r,s,n,\tilde{m},k:p\right]}\left(y_{1},y_{2}\right) & =f_{Y}\left(y_{1}\right)f_{Y}\left(y_{2}\right)\left[1+\Omega_{r,n,\tilde{m},k:p}\left(1-\left(1+p\right)F_{Y}^{p}\left(y_{1}\right)\right)\right.\\ & \left.+\Omega_{r,s,n,\tilde{m},k:p}\right.\times \left.\left(1-\left(1+p\right)F_{Y}^{p}\left(y_{2}\right)\right)\right.\\ & \left.+\Omega_{r,s,n,\tilde{m},k:p}^{⋆}\left(1-\left(1+p\right)F_{Y}^{p}\left(y_{1}\right)\right)\left(1-\left(1+p\right)F_{Y}^{p}\left(y_{2}\right)\right)\right], \end{array}$$

*where $\Omega_{r,s,n,\tilde{m},k:p}=\alpha \left[1-\left(1+p\right)C_{s-1}\left(\sum_{i=r+1}^{s}\frac{a_{i}^{\left(r\right)}\left(s\right)}{\gamma_{i}+p}\right)\left(\sum_{i=1}^{r}\frac{a_{i}\left(r\right)}{\gamma_{i}+p}\right)\right]$ and*

$$\begin{array}{c} \Omega_{r,s,n,\tilde{m},k:p}^{⋆}=\alpha \left[\Omega_{r,n,\tilde{m},k:p}-\alpha \left(1+p\right)\left(\sum_{i=r+1}^{s}\frac{a_{i}^{\left(r\right)}\left(s\right)}{\gamma_{i}+p}\right)\left(\sum_{i=1}^{r}\frac{a_{i}\left(r\right)}{\gamma_{i}+p}-\left(1+p\right)\sum_{i=1}^{r}\frac{a_{i}\left(r\right)}{\gamma_{i}+2p}\right)\right]. \end{array}$$

**Proof.**  By using (2) and (5), and the easy-to-prove relations $\sum_{i=r+1}^{s}\frac{a_{i}^{\left(r\right)}\left(s\right)}{\gamma_{i}}=\frac{C_{r-1}}{C_{s-1}}$ and $C_{r-1}\sum_{i=1}^{r}\frac{a_{i}\left(r\right)}{\gamma_{i}}=1,$ we get

$$\begin{array}{ccc} f_{\left[r,s,n,\tilde{m},k:p\right]}\left(y_{1},y_{2}\right) & = & \int_{-\infty }^{\infty }\int_{-\infty }^{x_{1}}\left[f_{Y}\left(y_{1}\right)\left(1+\alpha \left(1-\left(1+p\right)F_{X}^{p}\left(x_{1}\right)\right)\left(1-\left(1+p\right)F_{Y}^{p}\left(y_{1}\right)\right)\right)\right]\\ & \times & \left[f_{Y}\left(y_{2}\right)\left(1+\alpha \left(1-\left(1+p\right)F_{X}^{p}\left(x_{2}\right)\right)\left(1-\left(1+p\right)F_{Y}^{p}\left(y_{2}\right)\right)\right)\right]\\ & \times & C_{s-1}\left[\sum_{i=r+1}^{s}a_{i}^{\left(r\right)}\left(s\right){\left(\frac{F_{X}\left(x_{2}\right)}{F_{X}\left(x_{1}\right)}\right)}^{\gamma_{i}}\right]\left[\sum_{i=1}^{r}a_{i}^{}\left(r\right)F_{X}^{\gamma_{i}}\left(x_{1}\right)\right]\frac{f_{X}\left(x_{1}\right)}{F_{X}\left(x_{1}\right)}\frac{f_{X}\left(x_{2}\right)}{F_{X}\left(x_{2}\right)}dx_{2}dx_{1}\\ & = & C_{s-1}\int_{-\infty }^{\infty }\left[f_{Y}\left(y_{1}\right)\left(1+\alpha \left(1-\left(1+p\right)F_{X}^{p}\left(x_{1}\right)\right)\left(1-\left(1+p\right)F_{Y}^{p}\left(y_{1}\right)\right)\right)\right]\\ & \times & \left[\sum_{i=1}^{r}a_{i}^{}\left(r\right)F_{X}^{\gamma_{i}-1}\left(x_{1}\right)f_{X}\left(x_{1}\right)\right]\\ & \times & \left\{\int_{-\infty }^{x_{1}}\left[f_{Y}\left(y_{2}\right)\left(1+\alpha \left(1-\left(1+p\right)F_{X}^{p}\left(x_{2}\right)\right)\left(1-\left(1+p\right)F_{Y}^{p}\left(y_{2}\right)\right)\right)\right]\right.\\ & \times & \left.\left[\sum_{i=r+1}^{s}a_{i}^{\left(r\right)}\left(s\right){\left(\frac{F_{X}\left(x_{2}\right)}{F_{X}\left(x_{1}\right)}\right)}^{\gamma_{i}-1}\frac{f_{X}\left(x_{2}\right)}{F_{X}\left(x_{1}\right)}\right]dx_{2}\right\}dx_{1}\\ & = & \left[f_{Y}\left(y_{2}\right)\left(\sum_{i=r+1}^{s}\frac{a_{i}^{\left(r\right)}\left(s\right)}{\gamma_{i}}\right)+\alpha f_{Y}\left(y_{2}\right)\left(1-\left(1+p\right)F_{Y}^{p}\left(y_{2}\right)\right)\left(\sum_{i=r+1}^{s}\frac{a_{i}^{\left(r\right)}\left(s\right)}{\gamma_{i}}\right)\right]\\ & \times & \left.\left[C_{s-1}f_{Y}\left(y_{1}\right)\left(\sum_{i=1}^{r}\frac{a_{i}\left(r\right)}{\gamma_{i}}\right)\right.+\alpha C_{s-1}f_{Y}\left(y_{1}\right)\left(1-\left(1+p\right)F_{Y}^{p}\left(y_{1}\right)\right)\right.\\ & \times & \left.\left\{\left(\sum_{i=1}^{r}\frac{a_{i}\left(r\right)}{\gamma_{i}}\right)-\left(1+p\right)\left(\sum_{i=1}^{r}\frac{a_{i}\left(r\right)}{\gamma_{i}+p}\right)\right\}\right]\\ & - & \left[\left(1+p\right)\alpha f_{Y}\left(y_{2}\right)\left((1-\left(1+p\right)F_{Y}^{p}\left(y_{2}\right))\right)\sum_{i=r+1}^{s}\frac{a_{i}^{\left(r\right)}\left(s\right)}{\gamma_{i}+p}\right]\left[C_{s-1}f_{Y}\left(y_{1}\right)\left(\sum_{i=1}^{r}\frac{a_{i}\left(r\right)}{\gamma_{i}+p}\right)\right.\\ & + & \left.\alpha C_{s-1}f_{Y}\left(y_{1}\right)\left((1-\left(1+p\right)F_{Y}^{p}\left(y_{1}\right))\right)\left\{\left(\sum_{i=1}^{r}\frac{a_{i}\left(r\right)}{\gamma_{i}+p}\right)-\left(1+p\right)\left(\sum_{i=1}^{r}\frac{a_{i}\left(r\right)}{\gamma_{i}+2p}\right)\right\}\right]. \end{array}$$

 □

As a direct consequence of Theorem 2, the joint MGF of concomitants $Y_{\left[r,n,\tilde{m},k\right]}$ and $Y_{\left[s,n,\tilde{m},k\right]},r<s,$ in the HK–FGM model (3) is given by

(10) $$\begin{array}{cc} M_{\left[r,s,n,\tilde{m},k:p\right]}\left(t_{1},t_{2}\right) & =M_{Y}\left(t_{1}\right)M_{Y}\left(t_{2}\right)+\Omega_{r,n,\tilde{m},k:p}\left[M_{Y}\left(t_{1}\right)M_{Y}\left(t_{2}\right)-M_{V}\left(t_{1}\right)M_{Y}\left(t_{2}\right)\right]\;\\ & +\Omega_{r,s,n,\tilde{m},k:p}\left[M_{Y}\left(t_{1}\right)M_{Y}\left(t_{2}\right)-M_{Y}\left(t_{1}\right)M_{V}\left(t_{2}\right)\right]+\;\\ & \Omega_{r,s,n,\tilde{m},k:p}^{⋆}\left[M_{V}\left(t_{1}\right)-M_{Y}\left(t_{1}\right)\right]\left[M_{V}\left(t_{2}\right)-M_{Y}\left(t_{2}\right)\right]. \end{array}$$

The product moment $E\left[Y_{\left[r,n,\tilde{m},k\right]}^{\left(\ell \right)}Y_{\left[s,n,\tilde{m},k\right]}^{\left(q\right)}\right]=\mu_{\left[r,s,n,\tilde{m},k:p\right]}^{\left(\ell ,q\right)},\ell ,q>0$ is obtained directly from (10) as

(11) $$\begin{array}{ccc} \mu_{\left[r,s,n,\tilde{m},k:p\right]}^{\left(\ell ,q\right)} & = & \mu_{Y}^{\left(\ell \right)}\mu_{Y}^{\left(q\right)}+\Omega_{r,n,\tilde{m},k:p}\left[\mu_{Y}^{\left(\ell \right)}\mu_{Y}^{\left(q\right)}-\mu_{V}^{\left(\ell \right)}\mu_{Y}^{\left(q\right)}\right]+\Omega_{r,s,n,\tilde{m},k:p}\left[\mu_{Y}^{\left(\ell \right)}\mu_{Y}^{\left(q\right)}-\mu_{Y}^{\left(\ell \right)}\mu_{V}^{\left(q\right)}\right]\;\\ & + & \Omega_{r,s,n,\tilde{m},k:p}^{⋆}\left[\mu_{V}^{\left(\ell \right)}-\mu_{Y}^{\left(\ell \right)}\right]\left[\mu_{V}^{\left(q\right)}-\mu_{Y}^{\left(q\right)}\right]. \end{array}$$

**Remark** **2.** *By considering the well-known relation between the DGOSs and GOSs and by putting p=1 in (9), the JPDF of concomitants of GOSs for FGM can be easily deduced, which was obtained by [30].*

The following theorem gives a useful general recurrence relation for $\mu_{\left[r,s,n,\tilde{m},k:p\right]}^{\left(\ell ,q\right)}.$

**Theorem** **3.** *Let $L_{r}\left(p\right)=\sum_{i=1}^{r}\frac{a_{i}\left(r\right)}{\gamma_{i}+p}$ and $L_{r,s}\left(p\right)=\sum_{i=r+1}^{s}\frac{a_{i}^{\left(r\right)}\left(s\right)}{\gamma_{i}+p}.$ For any 1≤r<s−1≤n−1, we get*

$$\mu_{\left[r,s,n,\tilde{m},k:p\right]}^{\left(\ell ,q\right)}-\mu_{\left[r,s-1,n,\tilde{m},k:p\right]}^{\left(\ell ,q\right)}=\alpha p\left(1+p\right)C_{s-2}L_{r}\left(p\right)L_{r,s}\left(p\right)\left[\mu_{Y}^{\left(\ell \right)}\mu_{Y}^{\left(q\right)}-\mu_{Y}^{\left(\ell \right)}\mu_{V}^{\left(q\right)}\right]$$

$$-\alpha^{2}\left(1+p\right)\left(1-\gamma_{s}-p\right)\left(L_{r}\left(p\right)-\left(1+p\right)L_{r}\left(2p\right)\right)L_{r,s}\left(p\right)\left[\mu_{V}^{\left(\ell \right)}-\mu_{Y}^{\left(\ell \right)}\right]\left[\mu_{V}^{\left(q\right)}-\mu_{Y}^{\left(q\right)}\right].$$

**Proof.**  By using the easy-to-prove relations $a_{i}^{\left(r\right)}\left(s-1\right)=\left(\gamma_{s}-\gamma_{i}\right)a_{i}^{\left(r\right)}\left(s\right)$ and $\sum_{i=r+1}^{s}a_{i}^{\left(r\right)}\left(s\right)=0,$ we can easily get

$$\Omega_{r,s,n,\tilde{m},k:p}-\Omega_{r,s-1,n,\tilde{m},k:p}=-\alpha \left(1+p\right)L_{r}\left(p\right)\left[C_{s-1}L_{r,s}\left(p\right)-C_{s-2}L_{r,s-1}\left(p\right)\right]$$

$$=-\alpha \left(1+p\right)L_{r}\left(p\right)\frac{C_{s-1}}{\gamma_{s}}\sum_{i=r+1}^{s}\frac{\gamma_{i}a_{i}^{\left(r\right)}\left(s\right)}{\gamma_{i}+p}=\alpha p\left(1+p\right)C_{s-2}L_{r}\left(p\right)L_{r,s}\left(p\right)$$

and

$$\Omega_{r,s,n,\tilde{m},k:p}^{⋆}-\Omega_{r,s-1,n,\tilde{m},k:p}^{⋆}=-\alpha^{2}\left(1+p\right)\left(L_{r}\left(p\right)-\left(1+p\right)L_{r}\left(2p\right)\right)\left[L_{r,s}\left(p\right)-L_{r,s-1}\left(p\right)\right]$$

$$=-\alpha^{2}\left(1+p\right)\left(L_{r}\left(p\right)-\left(1+p\right)L_{r}\left(2p\right)\right)\sum_{i=r+1}^{s}\frac{\left(1-\gamma_{s}+\gamma_{i}\right)a_{i}^{\left(r\right)}\left(s\right)}{\gamma_{i}+p}$$

$$=-\alpha^{2}\left(1+p\right)\left(L_{r}\left(p\right)-\left(1+p\right)L_{r}\left(2p\right)\right)\left(1-\gamma_{s}-p\right)L_{r,s}\left(p\right).$$

By combining the two last equalities with (11), we get the required result. □

## 3. The Shannon Entropy for Concomitants of DGOSs from the HK–FGM Family

By using (6) and Theorem 2.2 in [10], we can easily get an explicit form of the Shannon entropy for concomitants of DGOSs from the HK–FGM model (3).

**Theorem** **4.** *If $Y_{\left[r,n,\tilde{m},k\right]}$ is a concomitants of rth DGOS in the HK–FGM family, then an explicit expression of the Shannon entropy of $Y_{\left[r,n,\tilde{m},k\right]},$ for 1≤r≤n, is given by*

(12) $$\begin{array}{c} H\left(Y_{\left[r,n,\tilde{m},k\right]}\right)\;=\;-E\left[\log f_{\left[r,n,\tilde{m},k:p\right]}\left(Y_{\left[r,n,\tilde{m},k\right]}\right)\right]\\ =\;Y_{r,n,\tilde{m},k:p}+H\left(Y\right)\left(1+\Omega_{r,n,\tilde{m},k:p}\right)+\left(1+p\right)\Omega_{r,n,\tilde{m},k:p}\Phi_{f:p}\left(u\right), \end{array}$$

*where $H\left(Y\right)=-E\left(\log f_{Y}\left(Y\right)\right)=-\int_{-\infty }^{\infty }f_{Y}\left(y\right)\log f_{Y}\left(y\right)dy$, i.e., the Shannon entropy of Y,$\Phi_{f:p}\left(u\right)=\int_{-\infty }^{\infty }F_{Y}^{p}\left(y\right)f_{Y}\left(y\right)\log f_{Y}\left(y\right)dy=\int_{0}^{1}u^{p}\log f_{Y}\left(F_{Y}^{-1}\left(u\right)\right)du$ and*

(13) $$\begin{array}{ccc} Y_{r,n,\tilde{m},k:p} & = & \frac{p^{2}\left(1+\Omega_{r,n,\tilde{m},k:p}\right)-p\Omega_{r,n,\tilde{m},k:p}}{1+p}-\log\left(1-p\Omega_{r,n,\tilde{m},k:p}\right)\\ & - & \frac{p(1+\Omega_{r,n,\tilde{m},k:p})}{1+p}\sum_{k=1}^{\infty }\frac{1}{k+\frac{1}{p}-1}{\left(\frac{\left(1+p\right)\Omega_{r,n,\tilde{m},k:p}}{1+\Omega_{r,n,\tilde{m},k:p}}\right)}^{k-1}. \end{array}$$

**Remark** **3.** *If $\Omega_{r,n,\tilde{m},k:p}\geq 0,$ representation (6) enables us to write*

$$\Omega_{r,n,\tilde{m},k:p}\leq \min_{\frac{1}{1+p}<F_{Y}^{p}\left(y\right)\leq 1}\left(\frac{1}{\left(1+p\right)F_{Y}^{p}\left(y\right)-1}\right)=\frac{1}{p}.$$

*Therefore, in order for the Shannon’s entropy defined in Theorem 4 to be finite, it is sufficient to assume that $p\Omega_{r,n,\tilde{m},k:p}\neq 1$ (this guarantees that $Y_{r,n,\tilde{m},k:p}$ is finite).*

In the next subsections, we study the moments and Shannon entropy of concomitants of DGOSs from the HK–FGM model (3) for some well-known DFs.

### 3.1. Exponential Distribution

The PDF and Df for exponential distribution are given by $f\left(y\right)=e^{-y},\;0\leq y<\infty ,$ and $F\left(y\right)=1-e^{-y}$, respectively. From (6), the PDF of the concomitant of $Y_{\left[r,n,\tilde{m},k\right]}$ is given by

$$f_{\left[r,n,\tilde{m},k:p\right]}\left(y\right)=e^{-y}\left[1+\Omega_{r,n,\tilde{m},k:p}\left(1-\left(1+p\right){\left(1-e^{-y}\right)}^{p}\right)\right].$$

Let $\mu_{\left[r,n,\tilde{m},k:p\right]}^{\left(\ell \right)}$ be the *ℓ*th moment of $Y_{\left[r,n,\tilde{m},k\right]}.$ Thus, from (8), we get

$$\mu_{\left[r,n,\tilde{m},k:p\right]}^{\left(\ell \right)}=\Gamma \left(\ell +1\right)\left[1+\Omega_{r,n,\tilde{m},k:p}\left(1-\left(1+p\right)\Delta \left(\ell ;p\right)\right)\right],$$

where $\Delta \left(\ell ;p\right)=\sum_{j=0}^{ℵ(p)}\frac{{\left(-1\right)}^{j}\left(\begin{array}{c} p\\ j \end{array}\right)}{{\left(j+1\right)}^{\ell +1}},$ℵ(p)=∞, if *p* is non-integer and ℵ(p)=p, if *p* is integer. From (9), the JPDF of $Y_{\left[r,n,\tilde{m},k\right]}$ and $Y_{\left[s,n,\tilde{m},k\right]}$ is given by

$$\begin{array}{c} f_{\left[r,s,n,\tilde{m},k:p\right]}\left(y_{1},y_{2}\right)=e^{-(y_{1}+y_{2})}\left[\begin{array}{c} 1+\Omega_{r,n,\tilde{m},k:p}\left[1-\left(1+p\right){\left(1-e^{-y_{1}}\right)}^{p}\right]+\Omega_{r,s,n,\tilde{m},k:p} \end{array}\right.\\ \times \left.\left[1-\left(1+p\right){\left(1-e^{-y_{2}}\right)}^{p}\right]+\Omega_{r,s,n,\tilde{m},k:p}^{⋆}\left[1-\left(1+p\right){\left(1-e^{-y_{1}}\right)}^{p}\right]\left[1-\left(1+p\right){\left(1-e^{-y_{2}}\right)}^{p}\right]\right]. \end{array}$$

Let $\mu_{\left[r,s,n,\tilde{m},k:p\right]}^{\left(\ell ,q\right)}$ be the *ℓ*th and *q*th joint moments of $Y_{\left[r,n,\tilde{m},k\right]}$ and $Y_{\left[s,n,\tilde{m},k\right]}.$ Thus, from (11) we get

$$\begin{array}{c} \mu_{\left[r,s,n,\tilde{m},k:p\right]}^{\left(\ell ,q\right)}=\Gamma \left(\ell +1\right)\Gamma \left(q+1\right)[1+\Omega_{r,n,\tilde{m},k:p}\left(1-\left(1+p\right)\Delta \left(\ell ;p\right)\right)+\Omega_{r,s,n,\tilde{m},k:p}\left(1-\left(1+p\right)\Delta \left(q;p\right)\right)\\ +\Omega_{r,s,n,\tilde{m},k:p}^{⋆}\left(1-\left(1+p\right)\Delta \left(\ell ;p\right)\right)\left(1-\left(1+p\right)\Delta \left(q;p\right)\right)]. \end{array}$$

**Theorem** **5.** *Let $Y_{\left[r,n,\tilde{m},k\right]}$ be the concomitant of rth DGOS for exponential distribution. Then, the Shannon’s entropy of $Y_{\left[r,n,\tilde{m},k\right]},$ for 1≤r≤n, is given by*

$$H\left(Y_{\left[r,n,\tilde{m},k\right]}\right)=Y_{r,n,\tilde{m},k:p}+\left(1+\Omega_{r,n,\tilde{m},k:p}\right)-\Omega_{r,n,\tilde{m},k:p}\left[\nu +\psi \left(p+2\right)\right],$$

*where $\nu =-\overset{´}{\Gamma }\left(1\right)=0.57722$ is the Euler’s constant, ψ(.) is the digamma function and $Y_{r,n,\tilde{m},k:p}$ is defined by (13).*

**Proof.**  From (12), we get

(14) $$\begin{array}{c} H\left(Y_{\left[r,n,\tilde{m},k\right]}\right)\;=\;Y_{r,n,\tilde{m},k:p}\;-\;\left(1+\Omega_{r,n,\tilde{m},k:p}\right)\int_{0}^{\infty }e^{-y}\log e^{-y}dy\;\\ +\left(1+p\right)\Omega_{r,n,\tilde{m},k:p}\int_{0}^{\infty }{\left(1-e^{-y}\right)}^{p}e^{-y}\log e^{-y}dy\;\\ =Y_{r,n,\tilde{m},k:p}+\left(1+\Omega_{r,n,\tilde{m},k:p}\right)+\left(1+p\right)\Omega_{r,n,\tilde{m},k:p}I. \end{array}$$

In order to find $I=\int_{0}^{\infty }{\left(1-e^{-y}\right)}^{p}e^{-y}\log e^{-y}dy,$ we note that $U\left(t\right)=\int_{0}^{\infty }{\left(1-e^{-y}\right)}^{p}$${\left[f\left(y\right)\right]}^{t}dy=\int_{0}^{\infty }{\left(1-e^{-y}\right)}^{p}e^{-ty}dy=\int_{0}^{1}{\left(1-z\right)}^{p}z^{t-1}dz=\beta \left(t,p+1\right)$ (by putting $z=e^{-y}$). Therefore, we get $\overset{´}{U}\left(t\right)=\frac{\partial U(t)}{\partial t}=\beta \left(t,p+1\right)\left[\psi (t)-\psi (p+1+t)\right].$ Thus, we get

(15) $$\overset{´}{U}\left(1\right)=I=\beta \left(1,p+1\right)\left[\psi (1)-\psi (p+2)\right]=\frac{1}{p+1}\left[\overset{´}{\Gamma }\left(1\right)-\psi \left(p+2\right)\right]=\frac{-1}{p+1}\left[\nu +\psi (p+2)\right].$$

By substituting (15) in (14), we get the required result. □

### 3.2. Pareto Distribution

The PDF and DF for Pareto distribution are given by $f\left(y\right)=ay^{-(a+1)},y\geq 1,$ and $F\left(y\right)=1-y^{-a},a>0$, respectively. From (6), the PDF of the concomitant of $Y_{\left[r,n,\tilde{m},k\right]}$ is given by

$$f_{\left[r,n,\tilde{m},k:p\right]}\left(y\right)=ay^{-(a+1)}\left[1+\Omega_{r,n,\tilde{m},k:p}\left(1-\left(1+p\right){\left(1-y^{-a}\right)}^{p}\right)\right].$$

Moreover, by using (8), the *ℓ*th moment of $Y_{\left[r,n,\tilde{m},k\right]}$ is given by

$$\mu_{\left[r,n,\tilde{m},k:p\right]}^{\left(\ell \right)}=\frac{a}{a-\ell }\left[1+\Omega_{r,n,\tilde{m},k:p}\left[1-\frac{\left(a-\ell )(p+1\right)}{a}\beta \left(\frac{a-\ell }{a},p+1\right)\right]\right],\;a>\ell .$$

From (9), the JPDF of $Y_{\left[r,n,\tilde{m},k\right]}$ and $Y_{\left[s,n,\tilde{m},k\right]}$ is given by

$$\begin{array}{c} f_{\left[r,s,n,\tilde{m},k:p\right]}\left(y_{1},y_{2}\right)=a_{1}a_{2}y_{1}^{-\left(a_{1}+1\right)}y_{2}^{-\left(a_{2}+1\right)}\left[1+\Omega_{r,n,\tilde{m},k:p}\left[1-\left(1+p\right){\left(1-y_{1}^{-a_{1}}\right)}^{p}\right]\right.\\ +\Omega_{r,s,n,\tilde{m},k:p}\times \left[1-\left(1+p\right){\left(1-y_{2}^{-a_{2}}\right)}^{p}\right]\\ +\Omega_{r,s,n,\tilde{m},k:p}^{⋆}\left[1-\left(1+p\right){\left(1-y_{1}^{-a_{1}}\right)}^{p}\right]\left[1-\left(1+p\right){\left(1-y_{2}^{-a_{2}}\right)}^{p}\right]. \end{array}$$

Finally, from (11), the *ℓ*th and *q*th joint moments of $Y_{\left[r,n,\tilde{m},k\right]}$ and $Y_{\left[s,n,\tilde{m},k\right]}$ are given by

$$\begin{array}{c} \mu_{\left[r,s,n,\tilde{m},k:p\right]}^{\left(\ell ,q\right)}=\frac{a_{1}a_{2}}{\left(a_{1}-\ell \right)\left(a_{2}-q\right)}\left[1+\Omega_{r,n,\tilde{m},k:p}\left[1-\frac{\left(a_{1}-\ell \right)\left(p+1\right)}{a_{1}}\beta \left(\frac{a_{1}-\ell }{a_{1}},p+1\right)\right]\right.\;\\ +\left.\Omega_{r,s,n,\tilde{m},k:p}\left[1-\frac{\left(a_{2}-q\right)\left(p+1\right)}{a_{2}}\beta \left(\frac{a_{2}-q}{a_{2}},p+1\right)\right]+\Omega_{r,s,n,\tilde{m},k:p}^{⋆}\right.\;\\ \times \left.\left[1\;-\;\frac{\left(a_{1}-\ell \right)\left(p+1\right)}{a_{1}}\beta \left(\frac{a_{1}-\ell }{a_{1}},p+1\right)\right]\;\;\left[1\;-\;\frac{\left(a_{2}-q\right)\left(p+1\right)}{a_{2}}\beta \left(\frac{a_{2}-q}{a_{2}},p+1\right)\right]\right],\\ a_{1}>\ell ,a_{2}>q. \end{array}$$

**Theorem** **6.** *Let $Y_{\left[r,n,\tilde{m},k\right]}$ be the concomitant of rth DGOS for Pareto distribution. Then, the Shannon entropy of $Y_{\left[r,n,\tilde{m},k\right]},$ for 1≤r≤n, is given by*

$$H\left(Y_{\left[r,n,\tilde{m},k\right]}\right)\;=\;Y_{r,n,\tilde{m},k:p}\;+\;\left(1+\Omega_{r,n,\tilde{m},k:p}\right)\left[1+\frac{1}{a}-\log a\right]\;-\;\Omega_{r,n,\tilde{m},k:p}\left[\nu +\psi (p+2)\;-\;\log a\right],$$

*where $Y_{r,n,\tilde{m},k:p}$ is defined by (13).*

**Proof.**  From (12), we get

(16) $$\begin{array}{c} H\left(Y_{\left[r,n,\tilde{m},k\right]}\right)=Y_{r,n,\tilde{m},k:p}-\left(1+\Omega_{r,n,\tilde{m},k:p}\right)\int_{1}^{\infty }ay^{-(a+1)}\log\left(ay^{-(a+1)}\right)dy\\ +\left(1+p\right)\Omega_{r,n,\tilde{m},k:p}\times \int_{1}^{\infty }{\left(1-y^{-a}\right)}^{p}ay^{-(a+1)}\log\left(ay^{-(a+1)}\right)dy\\ =Y_{r,n,\tilde{m},k:p}-\left(1+\Omega_{r,n,\tilde{m},k:p}\right)I_{1}+\left(1+p\right)\Omega_{r,n,\tilde{m},k:p}I_{2}. \end{array}$$

To find $I_{1}\;=\;-H\left(Y\right)\;=\;\int_{1}^{\infty }ay^{-(a+1)}\log\left(ay^{-(a+1)}\right)dy,$ we note that $T\left(t\right)\;=\;\int_{1}^{\infty }{\left[f\left(y\right)\right]}^{t}dy$$=\int_{1}^{\infty }a^{t}y^{-t(a+1)}dy$$=\frac{a^{t}}{t(a+1)-1}$ and $\frac{\partial T(t)}{\partial t}=\overset{´}{T}\left(t\right)=\frac{a^{t}\log a}{t(a+1)-1}-\frac{a^{t}\left(a+1\right)}{{\left(t\left(a+1\right)-1\right)}^{2}}.$ Thus,

(17) $$\begin{array}{c} \overset{´}{T}\left(1\right)=I_{1}=\log a-\frac{\left(a+1\right)}{a}. \end{array}$$

Furthermore, to find $I_{2}=\int_{1}^{\infty }{\left(1-y^{-a}\right)}^{p}ay^{-(a+1)}\log\left(ay^{-(a+1)}\right)dy.$ We note that $U\left(t\right)=\int_{1}^{\infty }{\left(1-y^{-a}\right)}^{p}{\left[f\left(y\right)\right]}^{t}dy$$=\int_{1}^{\infty }{\left(1-y^{-a}\right)}^{p}a^{t}y^{-t(a+1)}dy$$=a^{t-1}\int_{0}^{1}{\left(1-z\right)}^{p}z^{\frac{a+1}{a}\left(t-1\right)}dz$ = $a^{t-1}\beta \left(t\left(\frac{1+a}{a}\right)-\frac{1}{a},p+1\right)$ and

$$\overset{´}{U}\left(t\right)=a^{t-1}\beta \left(t\left(\frac{1+a}{a}\right)-\frac{1}{a},p+1\right)\left[\log a+\psi \left(t\left(\frac{1+a}{a}\right)-\frac{1}{a}\right)-\psi \left(t\left(\frac{1+a}{a}\right)-\frac{1}{a}+p+1\right)\right].$$

Therefore, we get

(18) $$\begin{array}{c} \overset{´}{U}\left(1\right)=I_{2}=\beta \left(1,p+1\right)\left[\log a+\psi (1)-\psi (p+2)\right]\;\\ =\frac{1}{p+1}\left[\log a+\overset{´}{\Gamma }\left(1\right)-\psi \left(p+2\right)\right]=\frac{-1}{p+1}\left[\nu +\psi (p+2)-\log a\right]. \end{array}$$

By substituting (17) and (18) in (16), we get the required result. □

### 3.3. Power Function Distribution

The PDF and DF for power distribution function are given by $f\left(y\right)=ay^{a-1},0\leq y\leq 1,$ and $F\left(y\right)=y^{a},a>0$, respectively. From (6) the PDF of the concomitant of $Y_{\left[r,n,\tilde{m},k\right]}$ is given by

$$f_{\left[r,n,\tilde{m},k:p\right]}\left(y\right)=ay^{a-1}\left[1+\Omega_{r,n,\tilde{m},k:p}\left(1-\left(1+p\right)y^{ap}\right)\right].$$

Moreover, from (8) the *ℓ*th moment of $Y_{\left[r,n,\tilde{m},k\right]}$ is given by

$$\mu_{\left[r,n,\tilde{m},k:p\right]}^{\left(\ell \right)}=\frac{a}{a+\ell }\left[1-\frac{p\ell \Omega_{r,n,\tilde{m},k:p}}{a(1+p)+\ell }\right].$$

Furthermore, from (9) the JPDF of $Y_{\left[r,n,\tilde{m},k\right]},Y_{\left[s,n,\tilde{m},k\right]},$ is given by

$$\begin{array}{c} f_{\left[r,s,n,\tilde{m},k:p\right]}\left(y_{1},y_{2}\right)=a_{1}a_{2}y_{1}^{a_{1}-1}y_{2}^{a_{2}-1}\left[1+\Omega_{r,n,\tilde{m},k:p}\left[1-\left(1+p\right)y_{1}^{a_{1}p}\right]\right.\\ +\Omega_{r,s,n,\tilde{m},k:p}\left[1-\left(1+p\right)y_{2}^{a_{2}p}\right]+\left.\Omega_{r,s,n,\tilde{m},k:p}^{⋆}\left[1-\left(1+p\right)y_{1}^{a_{1}p}\right]\left[1-\left(1+p\right)y_{2}^{a_{2}p}\right]\right], \end{array}$$

and from (11) the *ℓ*th and *q*th joint moments of $Y_{\left[r,n,\tilde{m},k\right]},Y_{\left[s,n,\tilde{m},k\right]},$ then:

$$\begin{array}{c} \mu_{\left[r,s,n,\tilde{m},k:p\right]}^{\left(\ell ,q\right)}=\frac{a_{1}a_{2}}{\left(a_{1}+\ell \right)\left(a_{2}+q\right)}\left[1-\frac{p\ell \Omega_{r,n,\tilde{m},k:p}}{a_{1}\left(1+p\right)+\ell }-\frac{pq\Omega_{r,s,n,\tilde{m},k:p}}{a_{2}\left(1+p\right)+q}\right.\\ \left.+\frac{p^{2}\ell q\Omega_{r,s,n,\tilde{m},k:p}^{⋆}}{\left(a_{1}\left(1+p\right)+\ell \right)\left(a_{2}\left(1+p\right)+q\right)}\right]. \end{array}$$

**Theorem** **7.** *Let $Y_{\left[r,n,\tilde{m},k\right]}$ be the concomitant of rth DGOS for power function distribution. Then, from (12), the Shannon entropy of $Y_{\left[r,n,\tilde{m},k\right]},$ for 1≤r≤n, is given by*

$$H\left(Y_{\left[r,n,\tilde{m},k\right]}\right)=Y_{r,n,\tilde{m},k:p}+\left(1+\Omega_{r,n,\tilde{m},k:p}\right)\left[1-\frac{1}{a}-\log a\right]-\Omega_{r,n,\tilde{m},k:p}\left[\frac{1}{1+p}-\frac{1}{a(1+p)}-\log a\right].$$

**Proof.**  From (12), we have

(19) $$\begin{array}{ccc} H(Y_{\left[r,n,\tilde{m},k\right]}) & = & Y_{r,n,\tilde{m},k:p}-\left(1+\Omega_{r,n,\tilde{m},k:p}\right)\int_{0}^{1}ay^{a-1}\log\left(ay^{a-1}\right)dy\\ & + & \left(1+p\right)\Omega_{r,n,\tilde{m},k:p}\int_{0}^{1}y^{ap}ay^{a-1}\log\left(ay^{a-1}\right)dy\\ & = & Y_{r,n,\tilde{m},k:p}-\left(1+\Omega_{r,n,\tilde{m},k:p}\right)I_{1}+\left(1+p\right)\Omega_{r,n,\tilde{m},k:p}I_{2}. \end{array}$$

To find $I_{1}=-H\left(Y\right)=\int_{0}^{1}ay^{a-1}\log\left(ay^{a-1}\right)dy,$ we note that $T\left(t\right)=\int_{0}^{1}{\left[f\left(y\right)\right]}^{t}dy=\int_{0}^{1}a^{t}y^{t(a-1)}dy$$=\frac{a^{t}}{t(a-1)+1}$ and $\frac{\partial T(t)}{\partial t}=\overset{´}{T}\left(t\right)$$=\frac{a^{t}\log a}{t(a-1)+1}-\frac{a^{t}\left(a-1\right)}{{\left(t\left(a-1\right)+1\right)}^{2}}.$ Thus, we get

(20) $$\begin{array}{c} \overset{´}{T}\left(1\right)=I_{1}=\log a-\frac{\left(a-1\right)}{a}. \end{array}$$

Furthermore, to find $I_{2}=\int_{0}^{1}y^{ap}ay^{a-1}\log\left(ay^{a-1}\right)dy,$ we note that $U\left(t\right)=\int_{0}^{1}y^{ap}{\left[f\left(y\right)\right]}^{t}$dy$=\int_{0}^{1}a^{t}y^{t(a-1)+ap}dy$$=\frac{a^{t}}{t(a-1)+ap+1}$ and $\overset{´}{U}\left(t\right)=\frac{a^{t}\log a}{t(a-1)+ap+1}-\frac{a^{t}\left(a-1\right)}{{\left(t\left(a-1\right)+ap+1\right)}^{2}}.$ Therefore, we get

(21) $$\begin{array}{c} \overset{´}{U}\left(1\right)=I_{2}=\frac{\log a}{1+p}-\frac{\left(a-1\right)}{a{\left(1+p\right)}^{2}}. \end{array}$$

By substituting (20) and (21) in (19), we get the required result. □

## 4. The FI Number for Concomitants of DGOSs from the HK–FGM Family

The FI number of the RV *X* having PDF $f_{X}\left(x\right)$ is defined by (cf. [40,41])

$$I_{{}_{f_{X}}}\left(X\right)=E{\left({\left.\frac{\partial \log f_{X}\left(x\right)}{\partial x}\right|}_{x=X}\right)}^{2}=\int_{-\infty }^{\infty }{\left(\frac{\partial f_{X}\left(x\right)}{\partial x}\right)}^{2}\frac{1}{f_{X}\left(x\right)}dx.$$

Clearly, the FI number has the following properties:

(I) $\;I_{{}_{f_{X}}}\left(cX\right)=I_{{}_{f_{X}}}\left(X\right)/c^{2},$ where c>0 is any scale parameter.
(II) $\;I_{{}_{f_{X+\theta }}}\left(X+\theta \right)=I_{{}_{f_{X}}}\left(X\right),$ where θ is any location parameter.

The property (II) expresses the well-known statement that the FI number is an FI for location parameter, or that the FI number is translation invariant. Moreover, by this property, we can suppress the possible dependence of $f_{X}$ on an unknown location parameter.

By using Theorem 2.3 in [10], we get an explicit form of FI number for concomitants of DGOSs from HK–FGM in the following theorem.

**Theorem** **8.** *Let $Y_{\left[r,n,\tilde{m},k\right]}$ be the concomitants of rth DGOS in the HK–FGM family. Then, the FI number of $Y_{\left[r,n,\tilde{m},k\right]},$ for 1≤r≤n, is given by*

(22) $$I_{{}_{f_{Y}}}\left(Y_{\left[r,n,\tilde{m},k\right]}\right)=I_{{}_{f_{Y}}}\left(Y\right)+\Omega_{r,n,\tilde{m},k:p}\left[\tau_{{}_{f_{Y}}}\left(p\right)-2p\left(1+p\right)\phi_{{}_{f_{Y}}}\left(p\right)\right]+{\left[\Omega_{r,n,\tilde{m},k:p}p\left(1+p\right)\right]}^{2}\delta_{f_{Y}}\left(p\right),$$

*where*

$$I_{{}_{f_{Y}}}\left(Y\right)=\int_{-\infty }^{\infty }{\left[\frac{\partial \log f_{Y}\left(y\right)}{\partial y}\right]}^{2}f_{Y}\left(y\right)dy,$$

$$\tau_{{}_{f_{Y}}}\left(p\right)=\int_{-\infty }^{\infty }{\left[\frac{\partial \log f_{Y}\left(y\right)}{\partial y}\right]}^{2}\left\{(1-\left(1+p\right)F_{Y}^{p}\left(y\right))\right\}f_{Y}\left(y\right)dy,$$

$$\phi_{{}_{f_{Y}}}\left(p\right)=\int_{-\infty }^{\infty }F_{Y}^{p-1}\left(y\right){\overset{´}{f}}_{Y}\left(y\right)f_{Y}\left(y\right)dy,$$

$$\delta_{{}_{f_{Y}}}\left(p\right)=\int_{-\infty }^{\infty }\frac{F_{Y}^{2(p-1)}\left(y\right)f_{Y}^{3}\left(y\right)dy}{1+\left(1-\left(1+p\right)F_{Y}^{p}\left(y\right)\right)\Omega_{r,n,\tilde{m},k:p}}.$$

In the next subsections, we will study the FI number for concomitants of DGOSs from HK–FGM for some well-known distributions such as exponential, Pareto and power function distributions.

### 4.1. Exponential Distribution

**Theorem** **9.** *Let $Y_{\left[r,n,\tilde{m},k\right]}$ be the concomitant of rth DGOS for exponential distribution. Furthermore, let $\Omega_{r,n,\tilde{m},k:p}>0,$$p\Omega_{r,n,\tilde{m},k:p}\neq 1,$ and $p>\frac{1}{2}.$ Then, the FI number of $Y_{\left[r,n,\tilde{m},k\right]},$ for 1≤r≤n, is given by*

(23) $$I_{{}_{f_{Y}}}\left(Y_{\left[r,n,\tilde{m},k\right]}\right)=1+2\Omega_{r,n,\tilde{m},k:p}+\frac{p^{2}{\left(1+p\right)}^{2}\Omega_{r,n,\tilde{m},k:p}^{2}}{1+\Omega_{r,n,\tilde{m},k:p}}\sum_{j=0}^{\infty }{\left(\frac{\left(1+p\right)\Omega_{r,n,\tilde{m},k:p}}{1+\Omega_{r,n,\tilde{m},k:p}}\right)}^{j}\beta \left(2p+jp-1,3\right).$$

**Proof.**  From (22), we get

(24) $$\begin{array}{cc} I_{{}_{f_{Y}}}\left(Y_{\left[r,n,\tilde{m},k\right]}\right) & =\int_{0}^{\infty }{\left[\frac{\partial \log f_{Y}\left(y\right)}{\partial y}\right]}^{2}\left\{1+\left(1-\left(1+p\right)F_{Y}^{p}\left(y\right)\right)\Omega_{r,n,\tilde{m},k:p}\right\}f_{Y}\left(y\right)dy-2p\left(1+p\right)\\ & \times \Omega_{r,n,\tilde{m},k:p}\int_{0}^{\infty }F_{Y}^{p-1}\left(y\right){\overset{´}{f}}_{Y}\left(y\right)f_{Y}\left(y\right)dy+{\left[\Omega_{r,n,\tilde{m},k:p}p\left(1+p\right)\right]}^{2}\\ & \times \int_{0}^{\infty }\frac{F_{Y}^{2(p-1)}\left(y\right)f_{Y}^{3}\left(y\right)dy}{1+\left(1-\left(1+p\right)F_{Y}^{p}\left(y\right)\right)\Omega_{r,n,\tilde{m},k:p}}=I_{1}+I_{2}+I_{3}, \end{array}$$

where

(25) $$\begin{array}{cc} I_{1} & =\int_{0}^{\infty }{\left[\frac{\partial \log f_{Y}\left(y\right)}{\partial y}\right]}^{2}\left\{1+\left(1-\left(1+p\right)F_{Y}^{p}\left(y\right)\right)\Omega_{r,n,\tilde{m},k:p}\right\}f_{Y}\left(y\right)dy\\ & =\int_{0}^{\infty }\left(1+\Omega_{r,n,\tilde{m},k:p}\right)e^{-y}dy-\left(1+p\right)\Omega_{r,n,\tilde{m},k:p}\int_{0}^{\infty }{\left(1-e^{-y}\right)}^{p}e^{-y}dy\\ & =\left(1+\Omega_{r,n,\tilde{m},k:p}\right)-\left(1+p\right)\Omega_{r,n,\tilde{m},k:p}\int_{0}^{1}u^{p}du=1, \end{array}$$

(26) $$\begin{array}{c} \begin{array}{cc} I_{2} & =-2p\left(1+p\right)\Omega_{r,n,\tilde{m},k:p}\int_{0}^{\infty }F_{Y}^{p-1}\left(y\right){\overset{´}{f}}_{Y}\left(y\right)f_{Y}\left(y\right)dy\\ & =2p\left(1+p\right)\Omega_{r,n,\tilde{m},k:p}\int_{0}^{\infty }{\left(1-e^{-y}\right)}^{p-1}e^{-2y}dy\\ & =2p\left(1+p\right)\Omega_{r,n,\tilde{m},k:p}\int_{0}^{1}u^{p-1}\left(1-u\right)du=2\Omega_{r,n,\tilde{m},k:p}, \end{array} \end{array}$$

$$I_{3}={\left[\Omega_{r,n,\tilde{m},k:p}p\left(1+p\right)\right]}^{2}\int_{0}^{\infty }\frac{F_{Y}^{2(p-1)}\left(y\right)f_{Y}^{3}\left(y\right)dy}{1+\left(1-\left(1+p\right)F_{Y}^{p}\left(y\right)\right)\Omega_{r,n,\tilde{m},k:p}}={\left(pd\right)}^{2}\int_{0}^{1}\frac{z^{2(p-1)}{\left(1-z\right)}^{2}dz}{c-dz^{p}}$$

(by using the transformation $z=1-e^{-y}$), $c=1+\Omega_{r,n,\tilde{m},k:p}$ and $d=\left(1+p\right)\Omega_{r,n,\tilde{m},k:p}.$ Now, in view of Remark 3, we have $0<p\Omega_{r,n,\tilde{m},k:p}<1.$ Thus, if 0≤z≤1, we get $0<\frac{d}{c}z^{p}<1.$ Therefore, by using the negative binomial expansion for ${\left(1-\frac{d}{c}z^{p}\right)}^{-1},$ we get

(27) $$I_{3}=\frac{{\left(pd\right)}^{2}}{c}\sum_{j=0}^{\infty }{\left(\frac{d}{c}\right)}^{j}\int_{0}^{1}z^{2(p-1)}{\left(1-z\right)}^{2}z^{jp}dz=\frac{{\left(pd\right)}^{2}}{c}\sum_{j=0}^{\infty }{\left(\frac{d}{c}\right)}^{j}\beta \left(2p+jp-1,3\right),$$

since $p>\frac{1}{2}.$ By substituting (25)–(27) in (24), the result follows. □

### 4.2. Pareto Distribution

**Theorem** **10.** *Let $Y_{\left[r,n,\tilde{m},k\right]}$ be the concomitant of rth DGOS for Pareto distribution. Furthermore, let $\Omega_{r,n,\tilde{m},k:p}>0,$$p\Omega_{r,n,\tilde{m},k:p}\neq 1,$ and $p>\frac{1}{2}.$ Then, the FI number of $Y_{\left[r,n,\tilde{m},k\right]},$ for 1≤r≤n, is given by*

$$\begin{array}{ccc} I_{{}_{f_{Y}}}\left(Y_{\left[r,n,\tilde{m},k\right]}\right) & = & \frac{a{\left(a+1\right)}^{2}\left(1+\Omega_{r,n,\tilde{m},k:p}\right)}{a+2}-{\left(a+1\right)}^{2}\left(1+p\right)\Omega_{r,n,\tilde{m},k:p}\;\beta \left(p+1,\frac{a+2}{a}\right)\\ & + & 2a^{}\left(a+1\right)p\left(1+p\right)\Omega_{r,n,\tilde{m},k:p}\beta \left(p,\frac{2a+2}{a}\right)\\ & + & \frac{a^{2}p^{2}{\left(1+p\right)}^{2}\Omega_{r,n,\tilde{m},k:p}^{2}}{1+\Omega_{r,n,\tilde{m},k:p}}\sum_{j=0}^{\infty }{\left(\frac{\left(1+p\right)\Omega_{r,n,\tilde{m},k:p}}{1+\Omega_{r,n,\tilde{m},k:p}}\right)}^{j}\beta \left(2p+jp-1,3+\frac{2}{a}\right). \end{array}$$

**Proof.**  From (22), we get

(28) $$\begin{array}{ccc} I_{{}_{f_{Y}}}\left(Y_{\left[r,n,\tilde{m},k\right]}\right) & = & \int_{1}^{\infty }{\left[\frac{\partial \ln f_{Y}\left(y\right)}{\partial y}\right]}^{2}\left\{1+\left(1-\left(1+p\right)F_{Y}^{p}\left(y\right)\right)\Omega_{r,n,\tilde{m},k:p}\right\}f_{Y}\left(y\right)dy-2p\left(1+p\right)\\ & \times & \Omega_{r,n,\tilde{m},k:p}\int_{1}^{\infty }F_{Y}^{p-1}\left(y\right){\overset{´}{f}}_{Y}\left(y\right)f_{Y}\left(y\right)dy+{\left[\Omega_{r,n,\tilde{m},k:p}p\left(1+p\right)\right]}^{2}\\ & \times & \int_{1}^{\infty }\frac{F_{Y}^{2(p-1)}\left(y\right)f_{Y}^{3}\left(y\right)dy}{1+\left(1-\left(1+p\right)F_{Y}^{p}\left(y\right)\right)\Omega_{r,n,\tilde{m},k:p}}=I_{1}+I_{2}+I_{3}, \end{array}$$

where

(29) $$\begin{array}{ccc} I_{1} & = & \int_{1}^{\infty }{\left[\frac{\partial \ln f_{Y}\left(y\right)}{\partial y}\right]}^{2}\left\{1+\left(1-\left(1+p\right)F_{Y}^{p}\left(y\right)\right)\Omega_{r,n,\tilde{m},k:p}\right\}f_{Y}\left(y\right)dy\\ & = & a{\left(a+1\right)}^{2}\left[\int_{1}^{\infty }\left(1+\Omega_{r,n,\tilde{m},k:p}\right)y^{-a-3}dy-\left(1+p\right)\Omega_{r,n,\tilde{m},k:p}\int_{1}^{\infty }{\left(1-y^{-a}\right)}^{p}y^{-a-3}dy\right]\\ & = & \frac{a{\left(a+1\right)}^{2}\left(1+\Omega_{r,n,\tilde{m},k:p}\right)}{a+2}-{\left(a+1\right)}^{2}\left(1+p\right)\Omega_{r,n,\tilde{m},k:p}\int_{0}^{1}u^{p}{\left(1-u\right)}^{\frac{2}{a}}du\\ & = & \frac{a{\left(a+1\right)}^{2}\left(1+\Omega_{r,n,\tilde{m},k:p}\right)}{a+2}-{\left(a+1\right)}^{2}\left(1+p\right)\Omega_{r,n,\tilde{m},k:p}\;\beta \left(p+1,\frac{a+2}{a}\right), \end{array}$$

(30) $$\begin{array}{ccc} I_{2} & = & -2p\left(1+p\right)\Omega_{r,n,\tilde{m},k:p}\int_{1}^{\infty }F_{Y}^{p-1}\left(y\right){\overset{´}{f}}_{Y}\left(y\right)f_{Y}\left(y\right)dy\\ & = & 2p\left(1+p\right)\Omega_{r,n,\tilde{m},k:p}\int_{1}^{\infty }{\left(1-y^{-a}\right)}^{p-1}a^{2}\left(a+1\right)y^{-2a-3}dy\\ & = & 2a^{}\left(a+1\right)p\left(1+p\right)\Omega_{r,n,\tilde{m},k:p}\int_{0}^{1}u^{p-1}{\left(1-u\right)}^{\frac{2}{a}+1}du\\ & = & 2a^{}\left(a+1\right)p\left(1+p\right)\Omega_{r,n,\tilde{m},k:p}\;\beta \left(p,\frac{2a+2}{a}\right), \end{array}$$

and (by using the transformation $z=1-y^{-a}$ and the abbreviations $c=1+\Omega_{r,n,\tilde{m},k:p}$ and $d=\left(1+p\right)\Omega_{r,n,\tilde{m},k:p}$)

$$\begin{array}{c} \begin{array}{c} I_{3}={\left[\Omega_{r,n,\tilde{m},k:p}p\left(1+p\right)\right]}^{2}\int_{0}^{\infty }\frac{F_{Y}^{2(p-1)}\left(y\right)f_{Y}^{3}\left(y\right)dy}{1+\left(1-\left(1+p\right)F_{Y}^{p}\left(y\right)\right)\Omega_{r,n,\tilde{m},k:p}}\\ =\frac{{\left(apd\right)}^{2}}{c}\int_{0}^{1}\frac{z^{2(p-1)}{\left(1-z\right)}^{2+\frac{2}{a}}dz}{1-\frac{d}{c}z^{p}}. \end{array} \end{array}$$

Now, in view of Remark 3, we have $0<p\Omega_{r,n,\tilde{m},k:p}<1.$ Thus, if 0≤z≤1, we get $0<\frac{d}{c}z^{p}<1.$ Therefore, by using the negative binomial expansion for ${\left(1-\frac{d}{c}z^{p}\right)}^{-1},$ we get

(31) $$\begin{array}{c} I_{3}=\frac{{\left(apd\right)}^{2}}{c}\sum_{j=0}^{\infty }{\left(\frac{d}{c}\right)}^{j}\int_{0}^{1}z^{2(p-1)}{\left(1-z\right)}^{2+\frac{2}{a}}z^{jp}dz\\ =\frac{{\left(apd\right)}^{2}}{c}\sum_{j=0}^{\infty }{\left(\frac{d}{c}\right)}^{j}\beta \left(2p+jp-1,3+\frac{2}{a}\right), \end{array}$$

since $p>\frac{1}{2}.$ By substituting (29), (30) and (31) in (28), the result follows. □

### 4.3. Power Function Distribution

**Theorem** **11.** *Let $Y_{\left[r,n,\tilde{m},k\right]}$ be the concomitant of rth DGOS for power function distribution. Furthermore, let $\Omega_{r,n,\tilde{m},k:p}>0,$$p\Omega_{r,n,\tilde{m},k:p}\neq 1,$ and a(2p+1)>2. Then, the FI number of $Y_{\left[r,n,\tilde{m},k\right]},$ for 1≤r≤n, is given by*

$$\begin{array}{ccc} I_{{}_{f_{Y}}}\left(Y_{\left[r,n,\tilde{m},k\right]}\right) & = & \frac{a{\left(a-1\right)}^{2}\left(1+\Omega_{r,n,\tilde{m},k:p}\right)}{a-2}-\frac{a\left(a-1\right)\left(1+p\right)\left[2ap+a-1\right]\Omega_{r,n,\tilde{m},k:p}}{(1+p)a-2}\\ & & +\frac{{\left(ap\left(1+p\right)\Omega_{r,n,\tilde{m},k:p}\right)}^{2}}{1+\Omega_{r,n,\tilde{m},k:p}}\sum_{j=0}^{\infty }\frac{{\left(\frac{\left(1+p\right)\Omega_{r,n,\tilde{m},k:p}}{1+\Omega_{r,n,\tilde{m},k:p}}\right)}^{j}}{2p-\frac{2}{a}+jp+1}. \end{array}$$

**Proof.**  From Equation (22), we have

(32) $$\begin{array}{cc} I_{{}_{f_{Y}}}\left(Y_{\left[r,n,\tilde{m},k\right]}\right) & =\int_{0}^{1}{\left[\frac{\partial \ln f_{Y}\left(y\right)}{\partial y}\right]}^{2}\left\{1+\left(1-\left(1+p\right)F_{Y}^{p}\left(y\right)\right)\Omega_{r,n,\tilde{m},k:p}\right\}f_{Y}\left(y\right)dy\\ & -2p\left(1+p\right)\times \Omega_{r,n,\tilde{m},k:p}\int_{1}^{\infty }F_{Y}^{p-1}\left(y\right){\overset{´}{f}}_{Y}\left(y\right)f_{Y}\left(y\right)dy\\ & +{\left[\Omega_{r,n,\tilde{m},k:p}p\left(1+p\right)\right]}^{2}\times \int_{0}^{1}\frac{F_{Y}^{2(p-1)}\left(y\right)f_{Y}^{3}\left(y\right)dy}{1+\left(1-\left(1+p\right)F_{Y}^{p}\left(y\right)\right)\Omega_{r,n,\tilde{m},k:p}}\\ & =I_{1}+I_{2}+I_{3}, \end{array}$$

where

(33) $$\begin{array}{cc} I_{1} & =\int_{0}^{1}{\left[\frac{\partial \ln f_{Y}\left(y\right)}{\partial y}\right]}^{2}\left\{1+\left(1-\left(1+p\right)F_{Y}^{p}\left(y\right)\right)\Omega_{r,n,\tilde{m},k:p}\right\}f_{Y}\left(y\right)dy\\ & =\int_{0}^{1}{\left(a-1\right)}^{2}y^{-2}\left[\left(1+\Omega_{r,n,\tilde{m},k:p}\right)-\left(1+p\right)\Omega_{r,n,\tilde{m},k:p}y^{ap}\right]ay^{a-1}dy\\ & =\frac{a{\left(a-1\right)}^{2}\left(1+\Omega_{r,n,\tilde{m},k:p}\right)}{a-2}-\frac{a{\left(a-1\right)}^{2}\left(1+p\right)\Omega_{r,n,\tilde{m},k:p}}{(1+p)a-2}, \end{array}$$

(34) $$\begin{array}{cc} I_{2} & =-2p\left(1+p\right)\Omega_{r,n,\tilde{m},k:p}\int_{0}^{1}F_{Y}^{p-1}\left(y\right){\overset{´}{f}}_{Y}\left(y\right)f_{Y}\left(y\right)dy\\ \; & =-2p\left(1+p\right)\Omega_{r,n,\tilde{m},k:p}\int_{0}^{1}y^{a(p-1)}a^{2}\left(a-1\right)y^{2a-3}dy\\ \; & =-2a^{2}{\left(a-1\right)}^{}p\left(1+p\right)\Omega_{r,n,\tilde{m},k:p}\int_{0}^{1}y^{(p+1)a-3}dy\\ \; & =\frac{-2a^{2}{\left(a-1\right)}^{}p\left(1+p\right)\Omega_{r,n,\tilde{m},k:p}}{(1+p)a-2}, \end{array}$$

and (by using the transformation $z=F\left(y\right)=y^{a}$ and the abbreviations $c=1+\Omega_{r,n,\tilde{m},k:p}$ and $d=\left(1+p\right)\Omega_{r,n,\tilde{m},k:p}$)

$$\begin{array}{cc} I_{3} & ={\left[p\left(1+p\right)\Omega_{r,n,\tilde{m},k:p}\right]}^{2}\int_{0}^{\infty }\frac{F_{Y}^{2(p-1)}\left(y\right)f_{Y}^{3}\left(y\right)dy}{1+\left(1-\left(1+p\right)F_{Y}^{p}\left(y\right)\right)\Omega_{r,n,\tilde{m},k:p}}\\ \; & =\frac{{\left(apd\right)}^{2}}{c}\int_{0}^{1}\frac{z^{2(p-1)}z^{2-\frac{2}{a}}dz}{1-\frac{d}{c}z^{p}}. \end{array}$$

Now, in view of Remark 3, we have $0<p\Omega_{r,n,\tilde{m},k:p}<1.$ Thus, if 0≤z≤1, we get $0<\frac{d}{c}z^{p}<1.$ Therefore, by using the negative binomial expansion for ${\left(1-\frac{d}{c}z^{p}\right)}^{-1},$ we get

(35) $$I_{3}=\frac{{\left(apd\right)}^{2}}{c}\sum_{j=0}^{\infty }\frac{{\left(\frac{d}{c}\right)}^{j}}{2p-\frac{2}{a}+jp+1},$$

since a(2p+1)>2. By substituting (33)–(35) in (32), the result follows. □

**Remark** **4.** *It is worth mentioning that some results in [38] are doubtful. For example, the FI number defined by (15) seems to be wrong, because we have $-1\leq \alpha C^{⋆}\left(r,n,m,k\right)\leq 1,$ which implies that $\log(-1+\alpha C^{⋆}\left(r,n,m,k\right))$ and $\log(-1-\alpha C^{⋆}\left(r,n,m,k\right))$ have no meaning, since $-2\leq -1+\alpha C^{⋆}\left(r,n,m,k\right)\leq 0$ and $-2\leq -1-\alpha C^{⋆}\left(r,n,m,k\right)\leq 0.$ Moreover, the integration defined in (19) seems to be wrong.*

## 5. Conclusions

In modelling bivariate data, when the prior information is in the form of marginal distributions, it is an advantage to consider families of bivariate distributions with specified marginals. Upon realizing that the HK–FGM provides a flexible and efficient family that can be used in such contexts, we studied the concomitants (which are related to the ordering bivariate RVs) of DGOSs (GOSs) under a general framework (when the parameters $\gamma_{1},\ldots ,\gamma_{n}$ are assumed to be pairwise different) from the HK–FGM family. We derived some useful relations that enable us to compute recursively the moments of the single and product of the concomitants of DGOSs for any arbitrary marginals (Theorems 1–3). The study of DGOSs (GOSs) under this framework enabled us to consider the POS model (Example 1) as an important censoring sampling scheme. In Section 3, we computed and studied the Shannon entropy for concomitants of DGOSs from the HK–FGM family (Theorem 4). Moreover, we computed the moments and Shannon entropy of concomitants of DGOS from the HK–FGM model for the exponential, Pareto and power function distributions (Theorems 5–7). The FI number was computed in Section 4, Theorem 8. Finally, the moments and FI number were computed in Theorems 9–11 for the exponential, Pareto and power function distributions, respectively.

Although all the results of this paper were obtained in a general framework, the only limitation of the adopted approach is perhaps the imperative of the bivariate data belonging to the HK–FGM DF. Clearly, by adopting the same procedure and the method of this paper, analogous results for other generalizations of the FGM model can be obtained.

When we talk about the prospects for future research, we consider here two problems. Estimation of the dependent parameters in HK–FGM DF or the associated parameters with the DF of the RV *Y* of primary interest using concomitants of DGOS or GOS values on the auxiliary RV *X* is an important future application. This problem has been tackled by [21] for *m*-GOSs and the generalized FGM family. The second future research problem is to use the kernel density estimator of the Shannon entropy defined in Theorem 4 (under the assumption that $f_{Y}$ is unknown). This estimator is the most commonly used nonparametric density estimator found in the literature (see, for example [42]).

## Data Availability

Not applicable.
